# Retinal Structure of *Poecilia sphenops*: Photoreceptor Mosaics, Synaptic Ribbon Patterns, and Glial Cell Expressions

**DOI:** 10.3390/ani14060939

**Published:** 2024-03-19

**Authors:** Doaa M. Mokhtar, Marco Albano, Rasha Alonaizan, Abdelraheim Attaai

**Affiliations:** 1 Department of Anatomy and Histology, Faculty of Veterinary Medicine, Assiut University, Assiut 71526, Egypt; doaa@aun.edu.eg; 2 Department of Anatomy and Histology, School of Veterinary Medicine, Badr University in Assiut, New Nasser City, Assiut 71515, Egypt; abdelraheim.attaai@aun.edu.eg; 3 Department of Veterinary Sciences, University of Messina, 98168 Messina, Italy; 4 Department of Zoology, College of Science, King Saud University, P.O. Box 2455, Riyadh 11451, Saudi Arabia; ralonezan@ksu.edu.sa; 5 Department of Anatomy and Embryology, Faculty of Veterinary Medicine, Assiut University, Assiut 71526, Egypt

**Keywords:** GFAP, rods, cones, photoreceptors, Müller cell, electron microscopy

## Abstract

**Simple Summary:**

This study scrutinized the retinal composition of Molly fish (*Poecilia sphenops*), revealing a complex neuronal structure. The retina showcased a distinctive square mosaic layout of cones, incorporating double cones alongside two variants of single cones. Within the inner nuclear layer, a diverse array of cells and Müller cell processes, expressing GFAP, traversed the retina. Notably, astrocyte cell processes expressing GFAP were discernible in both the inner and outer plexiform layers. Overall, Molly fish’s retina displayed a sophisticated structure characterized by a high density of photoreceptors, bipolar, amacrine, horizontal, Müller cells, and astrocytes, suggesting robust photopic visual capabilities.

**Abstract:**

The specific arrangement and distribution of photoreceptors in the retina can vary among different fish species, with each species exhibiting adaptations related to its habitat, behavior, and visual requirements. *Poecilia sphenops*, a diurnal fish, was the focus of this study. The retinas of a total of eighteen Molly fish were investigated utilizing light and electron microscopy. The retina exhibited a square mosaic pattern of the inner segments of cones. This pattern comprised double cones positioned along the sides of a square, with two types of single cones situated at the center and corners of the square arrangement across the entire retina. The corner cones were slightly shorter than the central ones. Additionally, the outer plexiform layer contained both cone pedicles and rod spherules. The rod spherule consisted of a single synaptic ribbon arranged in a triad or quadrat junctional arrangement within the invaginating free ends of the horizontal and bipolar cell processes. On the other hand, cone pedicles have more than one synaptic ribbon in their junctional complex. The inner nuclear layer consisted of the amacrine, bipolar, Müller, and horizontal cell bodies. Müller cell processes, expressing GFAP, extended across all retinal layers, segmenting the deeper retina into alternating fascicles of optic axons and ganglion cells. The outer and inner plexiform layers showed many astrocyte cell processes expressing GFAP. In conclusion, the current study is the first record of the retinal structures of Molly fish. This study illustrated the mosaic arrangement of photoreceptors and GFAP expression patterns of astrocytes and Müller cells. The presence of three cone types, coupled with a sufficient number of rods, likely facilitates motion awareness for tasks like finding food and performing elaborate mating ceremonies.

## 1. Introduction

The organization of the fish eye has the general structure that exists in higher vertebrates [1] in which three concentric layers make up each eye: an external fibrous layer composed of the white sclera and the translucent cornea, a middle vascular uveal layer, and the innermost layer of nervous tissue, the retina [2]. There are additional components in the eye such as the lens and the vitreous body, which are considered the refractive media of the eye. Almost all daylight fish possess color vision as good as that of normal humans [3].

Fish adapt functionally, morphologically, and evolutionarily to different light wavelengths in their habitats [4]. Several studies on various teleost species have demonstrated significant morphological heterogeneity, particularly in the light-sensitive part, the retina. This reflects adaptation to varied life habits, resulting in great differences in photoreceptor distribution, types, and densities [5]. Moreover, the differences include also the retinal pigment epithelia and their melanin content [6].

The position and size of the eye, the structure of the retinal photoreceptors, and the pigment epithelium influence vision in fish [7]. The feeding habits and photic environmental circumstances of the individual species are reflected in the interspecific variations in retinal anatomy. Rod cells have high visual sensitivity and are used in low-light conditions, whereas cone cells possess higher temporal and spatial resolution compared to rods and enable color vision and are more sensitive to several wavelengths [8]. Kunz [9] added that the middle- and long-wave sensitivity is subserved by the double cones, while short-wave sensitivity is achieved by the single cones.

In diurnal fishes, the retina is rich in cones, as indicated by the cone/rod ratio (photic sensitivity), while rod retinas are present primarily in nocturnal and deep-water fishes (scotopic sensitivity) [10]. Some deep-water fishes have thickened retinal layers, enabling the multiplying of rods to reach 20 million/mm^2^ [11]. The retina of teleost fish differs from the mammalian one in many aspects, such as having a highly regular mosaic pattern of photoreceptors and other retinal components, retino-motor movement as a light adaptation mechanism, and continuous growth throughout life [12].

There are four main varieties of cones: short, intermediate, long, and double. Occasionally, triple and quadruple cones are observed [9]. A cone photoreceptor mosaic pattern has been observed in a number of teleost species [13]. The mosaic pattern can differ in many different contexts, including between species, during various developmental stages, at different retinal sites, and even in response to environmental lighting [14]. The cone mosaic pattern is divided into three categories: row, square, and triangular. The row design features a classic cone pattern with parallel-oriented double cones. Double cone cells in the square pattern are alternately perpendicular at angles of 60° or 90°. In the triangular pattern, double cone cells are generally placed at angles of 60° and 120. In all mosaic forms, single cones are typically interspersed [15,16]. Notably, in the developmental stages of certain teleosts, there is a transition from a row pattern to a square pattern associated with a fish’s persistent migration from the surface (bright light) to deeper waters (dark light) [17].

The glial fibrillary acidic protein (GFAP) is the primary cytoskeletal intermediate filaments of glial cells in the vertebrates’ nervous system and is utilized as a marker for brain, retinal, and optic nerve astrocytes and the modified retinal astrocytes, the Müller cells [18]. GFAP immunohistochemistry was studied in restricted fish species, including zebrafish, goldfish, trout, and lizards [19,20,21,22].

*Poecilia sphenops* is one of the family Poeciliidae and is known as Molly. They live in freshwater streams but were also recorded in the coastal waters of Mexico [3]. Due to their diurnal nature, mollies are active during the day and sleep at night [23]. The Poeciliidae family is considered an ideal system for studying relationships between visual acuity and visual signals. They serve as a model system in the studying of mating choice, employing visual cues in both male–male competition and female mate selection. Colors or patterns, structural ornaments, or courting displays are examples of visual signals [24,25,26]. Despite the fact that visual signals in this family are diverse and important, little is known about their visual acuity.

Furthermore, while many papers exist elucidating the retina of the Guppy fish, there is a notable gap in the literature regarding the identification and arrangement of photoreceptors in Molly fish. This discrepancy served as a significant impetus for undertaking the current study on Molly fish. The primary objective of this research is to discern the types and organization of photoreceptors, alongside the topographical distribution of retinal neurons in Molly fish, employing histological, immunohistochemical, and electron microscopy analyses. This endeavor marks the first report detailing the retinal composition of Molly fish.

## 2. Materials and Methods

### 2.1. Samples Collection

The Ethics Committee of Assiut University in Egypt approved the study. The paired eyes of 18 Molly fish *(Poecilia sphenops*) were obtained from ornamental fish shops in Assiut City. The fish were housed in the laboratory under a 12 h light/dark cycle. All procedures were carried out in conformity with the applicable guidelines and ethical regulations. Aun/vet/4/0015 is the ethical number.

Before tissue sampling, fish were randomly selected from the aquariums and euthanized with an overdose of MS-222 (3% tricaine) [27]. The lens and the majority of the vitreous body were removed after the eyes were extracted and sliced open along the iris perimeter using an ophthalmic scissor. The retina was obtained by splitting the caudal half of the eyes slightly caudal to the iris.

### 2.2. Histological and Histochemical Analysis

The retinas were dissected quickly after euthanasia and were fixed in Bouin’s fluid for 22 h. The fixed specimens underwent ethanol dehydration, methyl benzoate clearing, and paraffin wax embedding. Serial longitudinal and transverse paraffin sections at 4–5 µm thickness using a microtome (Richert Leica RM 2125, Wetzlar, Germany) were cut and stained by Harris hematoxylin and Eosin. Alcian Blue stain (pH 2.5), Grimilus silver stain, and Iron hematoxylin stain were used for histochemical comparison [28].

### 2.3. Immunohistochemistry

For the immunostaining, 5 μm-thick retinal tissue sections were used after formalin fixation and paraffin embedding. The sections were boiled in 20 mL of Tris buffer and 2 mL of ethylene-diaminetetra acetic acid (pH 9.0) at 90 °C for 20 min. The sections were submerged in 3% H_2_O_2_ and then preincubated in 1% bovine serum albumin (B14, Thermo Fischer Scientific, Horsham, UK) in PBS for an entire night at 4 °C to block the endogenous peroxidase. The sections were stained using a rabbit polyclonal anti-glial fibrillary acidic protein (GFAP) [29] (1:500, PA5-16291, Thermo Fischer Scientific, Horsham, UK) for 30 min at room temperature based on the previously disclosed approach [30]. Hematoxylin-counterstained sections were inspected and photographed with a Leica microscope (Wetzlar, Germany).

### 2.4. Semithin Sections and TEM Preparations

The retinas were fixed by immersion in a combination of 2.5% paraformaldehyde–glutaraldehyde fixative for 24 h [31]. After fixation, the samples were washed in 0.1 Mol/L phosphate buffer and osmicated in 0.1 Mol/L sodium-cacodylate buffer at pH 7.3 with 1% osmium tetroxide. Then, the samples were dehydrated with ethanol, followed by propylene oxide, before being embedded in Araldite. Using Richert Ultracuts (Leica, Wetzlar, Germany), semithin sections (1 µm thick) were then stained with toluidine blue. Ultrotom VRV (LKB Bromma, Munich, Germany) was used to cut ultrathin sections. Sections measuring 70 nm were stained with a combination of lead citrate and uranyl acetate [32] and analyzed by JEOL 100CX II transmission electron microscope (JEOL, Tokyo, Japan) at Assiut University’s Electron Microscopy Unit.

### 2.5. Morphometrical Analysis

Using ImageJ software (v.1.54g), the densities of rods and cones were calculated by counting these visual cells in semithin sections of the retina. According to the methods of Hajar et al. and Hunt et al. [33,34], sections at the level of the double cone ellipsoids cross section were obtained. Three 100 µm transects were randomly selected from five different sections and images were captured under a light microscope at 400× magnification. The numbers of photoreceptor (PR) nuclei and cone ellipsoids were counted in each transect. Both double and single cones were counted as one unit. The following formula was employed to determine the number of rods:

Rods = PR nuclei − cone ellipsoids

Cell counts were expressed as density. The cellular density per CSA was performed by counting cells in the defined area and was finally calculated to 1 mm^2^. One-way ANOVA test followed by Tukey’s multiple range test was utilized to compare cone and rod densities in dorsal, ventral, temporal, and nasal retinal areas. Statistical significance was determined at *p* < 0.05. The statistical analysis was conducted using “GraphPad Software” (International Scientific Community, Version 6.05), with all data reported as means ± SE.

## 3. Results

### 3.1. Histological and Histochemical Analysis

**Figure 1:**
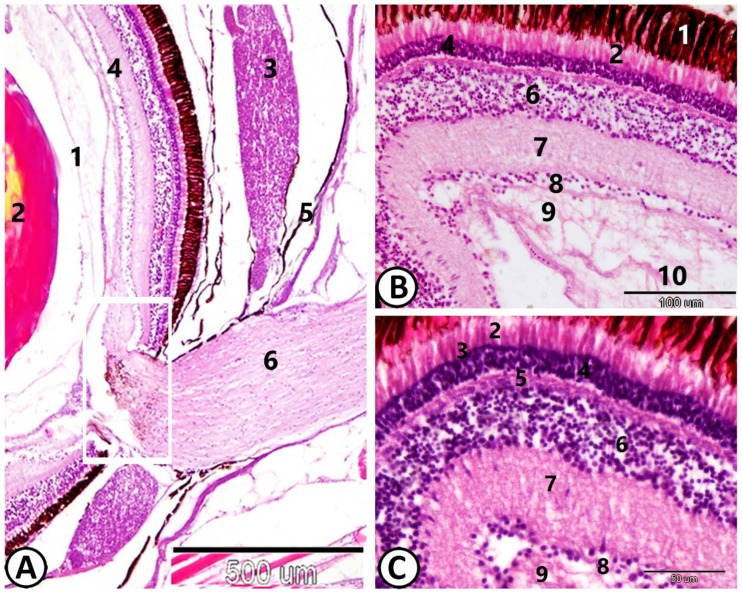
General structure of the eye of Molly fish stained with HE. (**A**) (1) Vitreous body. (2) The lens. (3) The choroid rete. (4) The retina. (5) The sclera. (6) Optic nerve (10). The white square indicating the optic disc. (**B**,**C**) Tangential section of the eye showing the layers of the retina. (1) The pigment epithelium, (2) the cones and rods, (3) ELM, (4) ONL, (5) OPL, (6) INL, (7) IPL, (8) the nuclei of the ganglion layer, (9) the optic nerve, (10) inner limiting membrane.

From an anatomical perspective, the eyes of Molly fish feature a sizable posterior chamber housing the vitreous body, along with anterior aqueous cavities separated by the lens. The retina lies internally to the choroid. The optic nerve appeared clearly distinguishable (Figure 1A). The retina (Figure 1B,C) was composed of ten successive layers: (1) the pigment epithelium; (2) the rods and cones; (3) the external limiting membrane (ELM); (4) the outer nuclear layer (ONL) containing the photoreceptors’ cell bodies; (5) the outer plexiform layer (OPL); (6) the inner nuclear layer (INL); (7) the inner plexiform layer (IPL); (8) the ganglion layer, (9) the optic nerve fiber layer, and (10) the inner limiting membrane (ILM), which consists of the extended processes of the glial cells.

**Figure 2:**
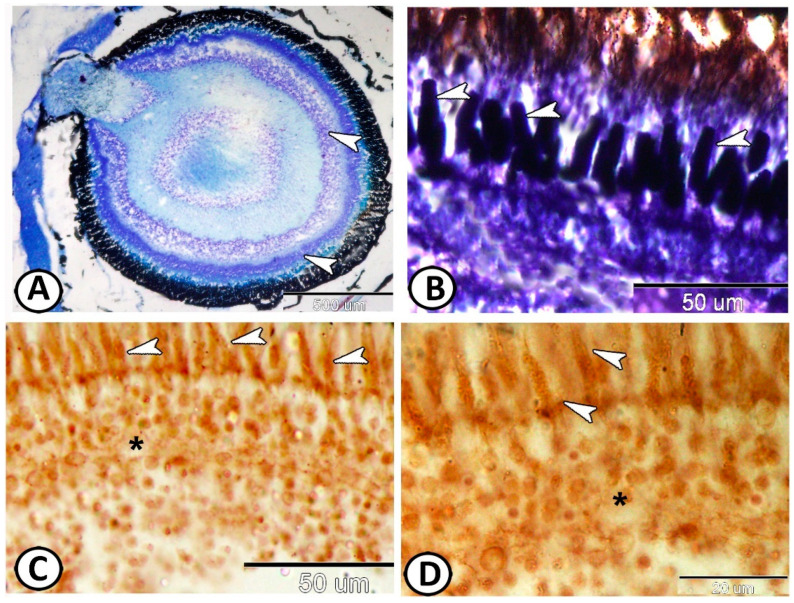
Histochemical analysis: (**A**) AB-positive outer and inner plexiform layers of the retina (arrowheads). (**B**) The mitochondria in photoreceptors showed a strong reaction to Iron hematoxylin (arrowheads). (**C**,**D**) Grimilus silver stain showing argyrophilic properties of neuronal cell bodies and axonal processes of photoreceptors (arrowheads) and outer nuclear layer (asterisk).

The histochemical analysis indicated that both plexiform layers of the Molly fish retina showed positive reactions for alcian blue (Figure 2A). In addition, the mitochondria of the photoreceptors showed a strong reaction to Iron hematoxylin staining (Figure 2B). Grimlius silver staining showed the argyrophilic properties of neuronal cell bodies and the axonal processes of photoreceptors and the outer nuclear layer (Figure 2C,D).

**Figure 3:**
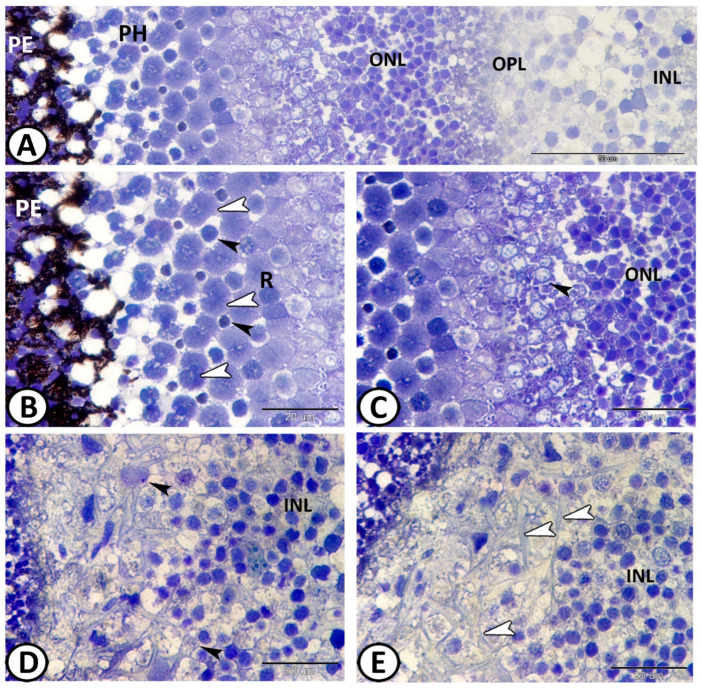
Semithin section through the retina stained with TB. (**A**) Pigmented epithelium (PE) was followed by photoreceptors (PH), outer nuclear layer (ONL), outer plexiform layer (OPL), and inner nuclear layer (INL). (**B**) Mosaic pattern of inner segments of cones showing double cones (white arrowheads) surrounding single cones (black arrowheads). Note the processes of pigmented epithelium (PE). The rods (R). (**C**) The cone/rod myoids (arrowhead) and outer nuclear layer (ONL). (**D**,**E**) The outer plexiform layer showing horizontal cells (arrowheads) in the outer region of inner nuclear layer (INL).

Molly fish retinas were duplex, with cones and rods located in a single layer (Figure 3B,C). The retina was arranged into a square mosaic pattern of the inner segments of cones. This pattern comprised double cones positioned along the sides of a square, with two types of single cones situated at the center and corners of the square arrangement across the entire retina. The corner cones were slightly shorter than the central ones (Figure 3B).

The semithin sections showed that the photoreceptors and the retinal pigment epithelium were next to each other. Many elongated processes emanated from the apical end of the epithelial cells and extended towards the inner segment of cones and rods. Melanin pigments were hosted in the cytoplasm of the epithelial cells (Figure 3A,B). Molly fish had duplex retinas, meaning that rods and cones were arranged in a single layer (Figure 3B,C). Horizontal cells were situated in the outer region of the INL. They displayed irregular cell bodies that sent long horizontal processes in this layer (Figure 3D,E).

**Figure 4:**
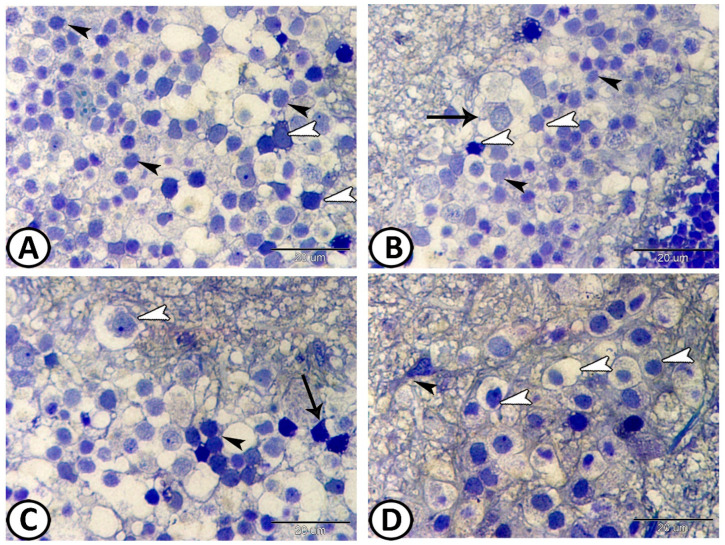
Semithin sections through the retina stained with TB. (**A**,**B**) Inner nuclear layer consisted of nuclei of amacrine (white arrowheads) and bipolar cells (black arrowheads). Note the horizontal cells (arrow). (**C**) IPL consisted of processes of amacrine (arrow), bipolar cells (black arrowheads), and ganglion cells (white arrowheads). (**D**) The ganglionic layer contained ganglion cells (white arrowheads) and astrocytes (black arrowhead).

The INL contained nuclei of amacrine and bipolar cells (Figure 4A,B). The amacrine were stellate cells located around the bipolar cells that appeared spherical in shape. The IPL consisted of processes of the bipolar, amacrine, and ganglionic cells (Figure 4C). The ganglion cell layer consisted of large ganglion cells with rounded nuclei and astrocytes (Figure 4D).

#### Immunohistochemistry

**Figure 5:**
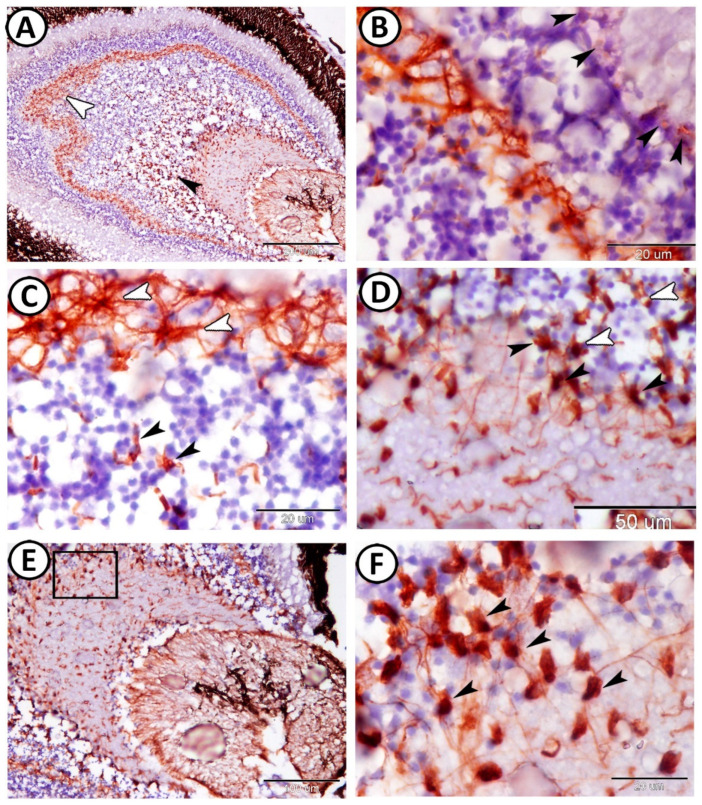
GFAP expression in the retina of Molly. (**A**) GFAP immunoreactive Müller cells were distributed within OPL (white arrowhead) and the outer zone of the IPL (black arrowhead). (**B**) GFAP immunoreactive small extensions of Müller cell processes (arrowheads) were observed between the inner segments of the photoreceptors. (**C**) The astrocytes with unique cell processes expressed GFAP in OPL (white arrowheads) and inner nuclear layer (black arrowheads). (**D**) There are many short processes that emerge from Müller cells’ bodies in INL (white arrowheads) toward the ganglion layer (black arrowheads). (**E**,**F**) GFAP immunoreactive thick processes extended from the Müller cells and appeared next to the ganglion layer (boxed area, arrowheads).

GFAP immunoreactive cells, Müller cells, were distributed within the inner territories of the inner nuclear, OPL, and the outer zone of the IPL (Figure 5A). GFAP immunoreactive puncta (small extensions) of Müller cell processes were observed between the inner segments of the cones and rods (Figure 5B). Astrocytes with unique cell processes expressed GFAP in the OPL and INL (Figure 5C). Many short processes emerge from Müller cells’ bodies in the INL (Figure 5D). GFAP immunoreactive thick processes appeared next to the ganglion cell layer (Figure 5E). Moreover, thin branches extended from the Müller cells in the ganglionic layer. Most of these branches were directed towards the inner limiting membrane layer (Figure 5F).

### 3.2. Transmission Electron Microscopy

#### The Cellular Constituents of the Retina Include

**Figure 6:**
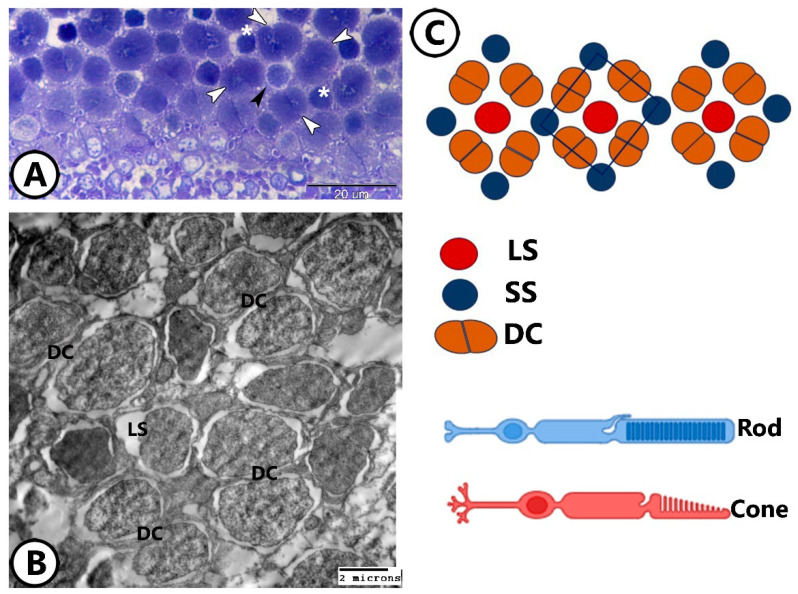
The mosaic pattern of the cone photoreceptors. (**A**,**B**) Semithin and TEM images show a square mosaic pattern of the inner segments of cones. This pattern showing double cones (DCs, white arrowheads) and two types of single cones, in which double cones occupy the sides of a square, and long single cones (LSs, black arrowhead) occupy the center, and the short single cone (SS, asterisk) present in the corners of the square pattern along the entire retina. (**C**) Schematic diagram of a section through the ellipsoid region of the cone inner segments showing the square mosaic pattern of the retina. LS: Long single cone, SS: Short single cone, DCs: Double cones. Note the single cones at the corners and the center of the square units with the double cones forming the sides.

**Figure 7:**
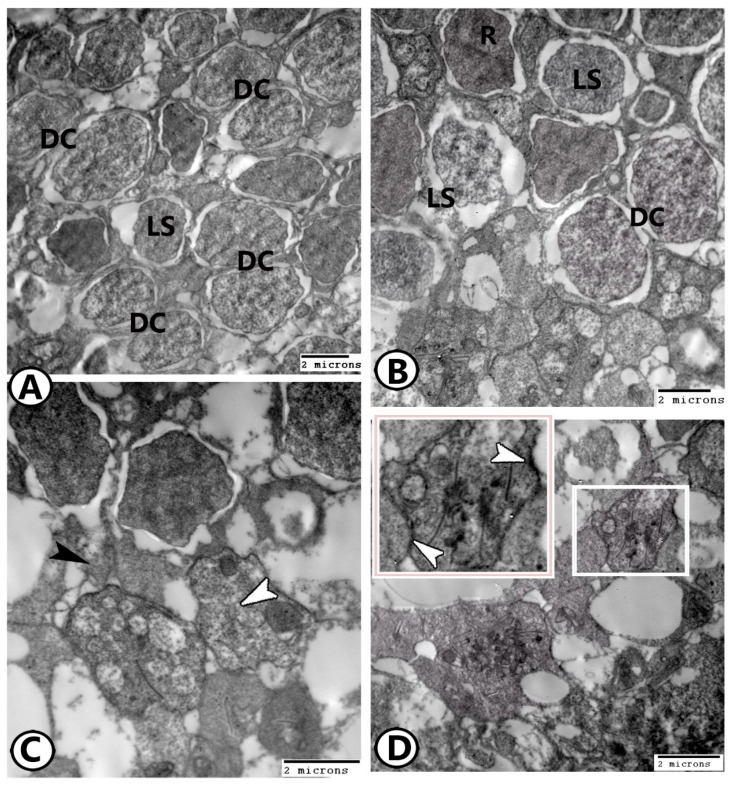
TEM images of photoreceptors (**A**,**B**): The photoreceptors showing the mosaic pattern of double cones (DCs) and long single central cones (LSs). Rods (R). (**C**) In the OPL, a pedicle (black arrowhead) with lateral extensions of rods that make ribbon–synaptic connections (white arrowhead) with other neurons. (**D**) The second synaptic type shows dark parts of adjacent cell membranes of the photoreceptors (arrowheads in boxed area).

*(1) The photoreceptor cells:* The photoreceptors included four types: double cones, single long cones, single short cones, and rods. Most cones were double cones (DCs) (Figure 6A–C and Figure 7A). Double cones are partially fused at their inner segments. Both are symmetrical in shape and have the same color. However, they are unequal in length. Their nuclei were euchromatic and lay immediately under the OLM, in the ONL.

Different cones are arranged regularly in a mosaic pattern (Figure 7B). The cell extensions of photoreceptors reach the OPL. Their free end has a pedicle with circumference extensions that make synapses with other neurons. Two types of synapses could be recognized: the first was ribbon synapses, which were established between the interdigitation (ribbon) of both bipolar and horizontal cells, and the ends of the photoreceptor cells (Figure 7C). The second type was recognized by the dark parts of the adjacent cell membranes of the cones and rods (Figure 7D).

**Figure 8:**
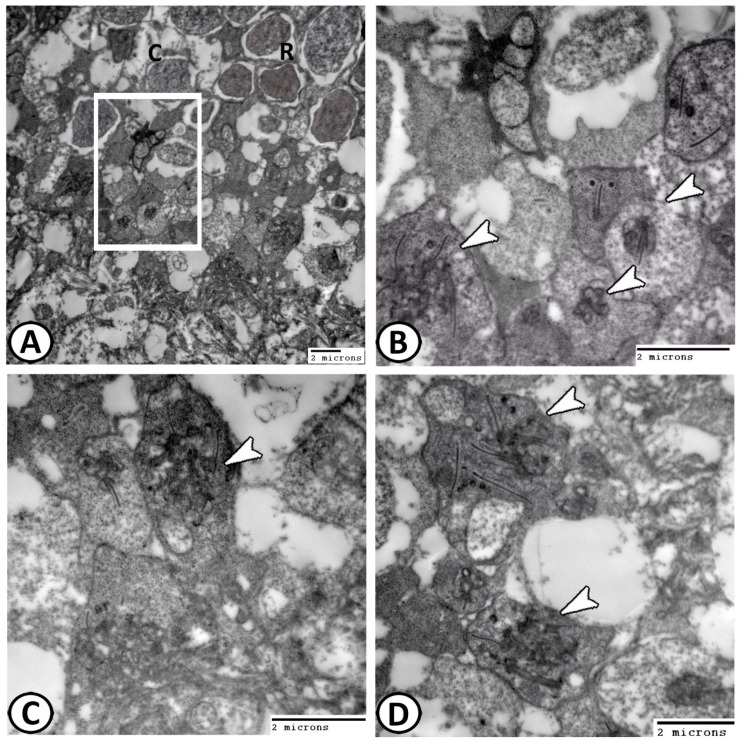
TEM images of photoreceptors (**A**): The rods (R) showed uniform size and shape of outer and inner segments. The cones (C). (**B**) Higher magnification of the square area in Figure A showing that the cones have pedicles with many synaptic ribbons (arrowheads). (**C**,**D**) Rods displayed spherule synaptic terminals with a single synaptic ribbon (arrowheads).

**Figure 9:**
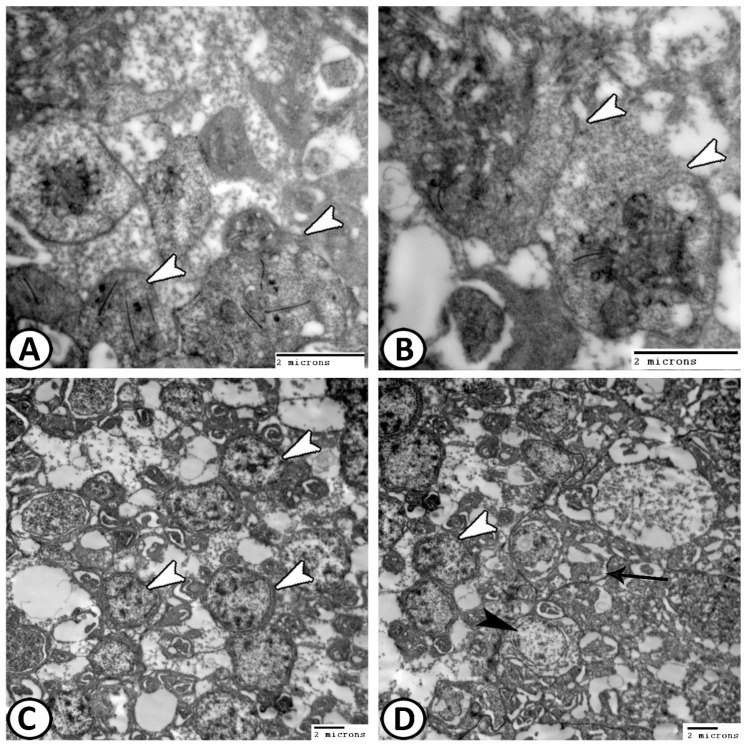
TEM of the OPL and IPL: (**A**,**B**) The outer plexiform layer showing extensive synaptic connections (arrowheads). (**C**) Bipolar cells (arrowheads) were spherical neurons. (**D**) IPL contained the synapses between bipolar (white arrowhead), amacrine cells (black arrowhead), and ganglion cells (arrow).

The rods showed uniform shapes and sizes of inner and outer segments. The nuclei were heterochromatic and located deep in the ONL (Figure 8A). Both rods and cones were identified by their synaptic terminals. The cones have pedicles with many synaptic ribbons (Figure 8B), while rods display spherule synaptic terminals (Figure 8C,D). In the transverse view, the complicated arrangement of invaginations contains a single synaptic ribbon of rod spherules (Figure 8C), whereas cone pedicles bear 3–4 synaptic ribbons (Figure 8B). Notably, the outer plexiform layer exhibited extensive synaptic connections (Figure 9A,B).

The morphometrical analysis (Table 1) indicated that the density of cones per square mm (mm^2^) was as follows: 7143 dorsally, 7370 temporally, 8786 ventrally, and 6733 nasally (Table 1). The density of rods per mm^2^ was 6112 dorsally, 5679 temporally, 5832 ventrally, and 4932 nasally.

*(2) Bipolar cells:* These neurons have spherical dense nuclei. Bipolar cells were radially oriented with lower terminal branches in the IPL and dendrite branches in the OPL, which contact the rods and cones (Figure 9C). The IPL contained the synapses between the bipolar and amacrine cells and the ganglion cells. In this layer, both the synaptic vesicles and the extensions of the aforementioned cells were seen (Figure 9D).

**Figure 10:**
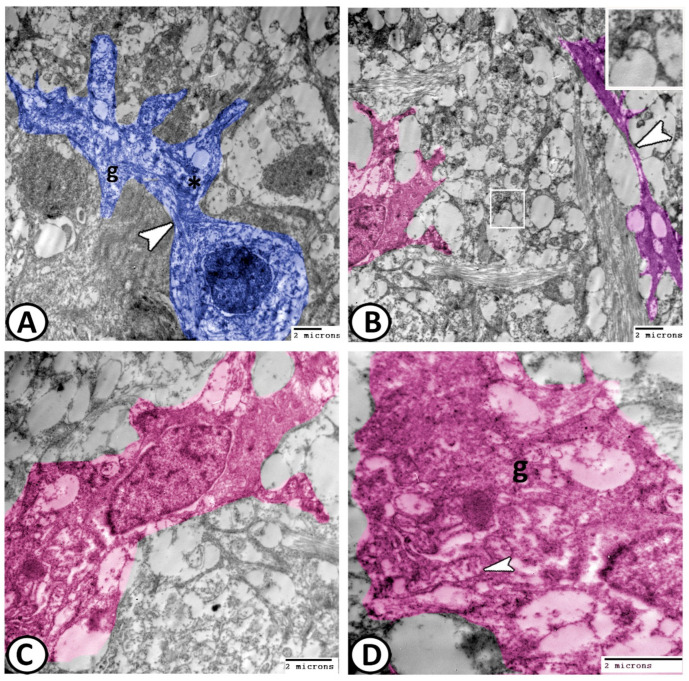
Digital colored image of horizontal and amacrine cells: (**A**) horizontal cells (blue) with finger-like process. The cytoplasm shows many glycogen granules (g) and dense bodies (asterisk). Numerous parallel fibril bundles run within the lateral expansions (arrowhead). (**B**) INL involves the amacrine nuclei (pink), which extended their processes to Müller cells in the inner plexiform layer (violet colored, white arrowhead). Note the astrocytes (boxed areas). (**C**,**D**) Amacrine cells show a slightly euchromatic nucleus, mitochondria (arrowhead), and glycogen granules (g).

*(3) Horizontal cells:* These cells were seen in the outer region of the INL. They had finger-like processes that extended horizontally, reaching the OPL and synapses with rod and cone invaginations. Their cytoplasm showed abundant mitochondria, glycogen granules, and electron-dense bodies. Numerous parallel fibrils bundles ran within the lateral expansions (Figure 10A). The INL involves the nuclei of the amacrine and bipolar cells, which extended their processes to the IPL (Figure 10B).

*(4) Amacrine cells:* These stellate cells are located between the INL and IPL and showed a slightly euchromatic nucleus, mitochondria, and glycogen granules (Figure 10C,D).

**Figure 11:**
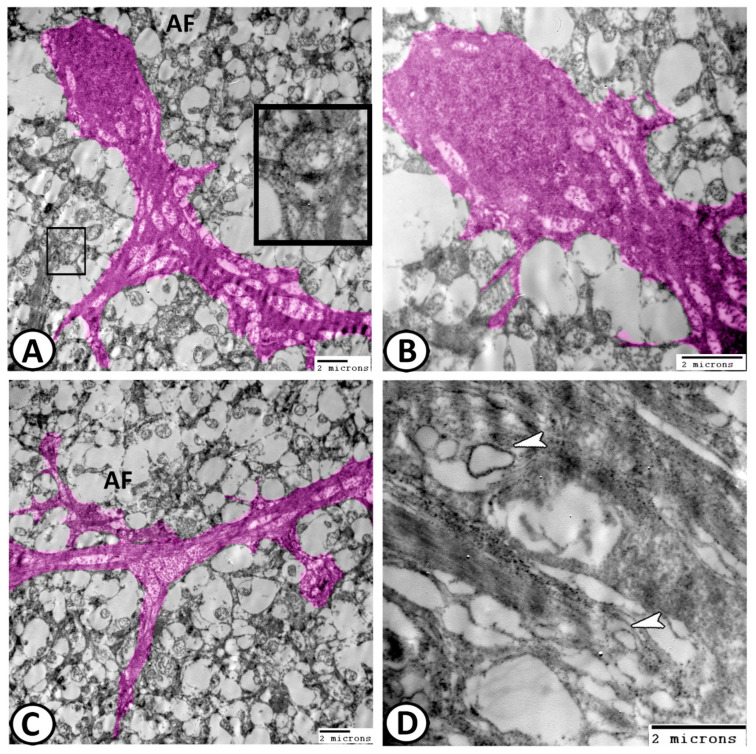
Digital colored image of Müller cells. (**A**–**C**) Thick Müller cell processes (pink) divide two axon fascicles (AF). Note the inserted figure in A is amacrine cell. (**D**) The optic nerve layer consisted of Ganglion cell axons (arrowheads).

*(5) Müller cells* are the retinal specialized astrocytes. They started at the basal lamina that forms the ILM and extends towards the OLM where they contact the photoreceptors through cellular junctions. Their nuclei were heterochromatic and placed in the ONL (Figure 11A–C). The optic nerve layer consisted of the axons of Ganglion cells, which were predominantly unmyelinated; however, rare myelinated profiles were sometimes seen (Figure 11D).

## 4. Discussion

The general morphology and structure of the eye are largely conserved in vertebrates; however, there are morphologic differences between different parts, such as the choroid and retina, in different classes [35]. Even between different species of fish, a significant morphological difference was evident [36].

The cones exhibited variations in type, density, shape, and size among different fish species [3]. Color vision demands the existence of several cone photopigments. Teleost fish exhibit tri- or dichromatic vision. Certain species like brown trout and some cyprinids display tetrachromacy, characterized by the presence of UV-sensitive cones arranged in a distinct mosaic pattern [37,38]. The loss of UV-sensitivity observed in the 2 years old trout corresponded with the disappearance of the corner cones (cc) within the square cone mosaic. Consequently, it was concluded that the cc represents the UV-sensitive cells [37]. In older salmonids, where the cc are lost in the main retina, the potential UV cones persistently develop within two highly defined, narrow growth zones. However, these cones are subsequently lost before integrating into the main retina [37]. The present study supported the results of Kunz [15] in an ecologically related species, *Poecilia reticulata*, that the photoreceptors of Molly fish are arranged in a square mosaic pattern. Donatti and Fanta [4] reported that most diurnal species living in shallow water displayed a mosaic appearance of double cones and single ones. In addition, Kunz [15] proved that the square mosaic pattern of retinal cones changes during dark adaptation into a row mosaic in guppy, *Poecilia reticulata*. The square mosaic pattern likely enhances motion detection, facilitating activities such as locating food and engaging in intricate mating rituals. Compared to row mosaics, square mosaics offer the advantage of perceiving movement from all directions. In the eye, regions featuring row mosaics are typically associated with dim vision, whereas those with square mosaics are specialized for acute vision [39]. The mosaic patterns allow for gathering the moving visual stimuli [40]. Another closely related proposed role for a regular mosaic incorporating double cones is the polarized light detection that would be useful in navigating and detecting prey in turbid water [41].

Multiple cone types have been found in cartilaginous and early ray-finned fish (three cone types), dipnoan (four cone types), and teleosts (up to seven cone types) [42,43,44,45]. As a result, all fish groups have the possibility of color vision, albeit this has yet to be behaviorally proven in non-actinopterygian species [46].

Another mosaic configuration observed is the row mosaic, identified in the retina of adult zebrafish (*Danio rerio*), characterized by alternating rows of single cones and double cones [47]. Certain species, including atheriniforms, osmerids, and salmonid fishes [48,49], display pentagonal and hexagonal arrangements where five and six double cones, respectively, surround a single cone [49,50]. Additionally, the shapes of double cones can vary along their lengths, as seen in salmonid fishes [51]. Salmonids also have specialized cones, which are intensely sensitive to ultraviolet light [52].

Catfish, which inhabit turbid environments and predominantly depend on nonvisual cues for locating prey, possess only single cones within their retinas [53]. Furthermore, numerous fish species with grouped retinae lack cone mosaics with double cones. Instead, in these species, single cones or rods are grouped to form “megareceptors” whose primary function is to improve overall sensitivity and, consequently, contrast based on luminosity [54]. These fish inhabit extremely low-light environments and typically do not depend on high-acuity vision for prey localization. Among bathypelagic fishes, which frequently reside at depths exceeding 1000 m [55], most lack cones and instead have multiple banks of rods [55,56]. These retinas are adapted for maximum sensitivity rather than visual acuity. In certain deep-water fishes, tubular eyes are observed, featuring a fovea where double cones align in rows, hypothesized to facilitate motion detection [57]. The absence of square mosaics in deep water fishes indirectly suggests a potential role for such mosaic types in visual acuity and possibly color contrast.

Photoreceptor tips exhibit a diurnal cycle, with cones shed at night and rods shed throughout the day [57]. The guppy *P. reticulata* cone-IS contains ellipsosomes, which are intracellular color filters. They are mitochondrial in origin and operate similarly to colored oil droplets found in birds and reptiles. Instead of oil, they include the heme pigment cytochrome C, which improves color contrast in the blue-violet range [58].

Twin cones are frequent in several bony fishes. The ratio of these photoreceptive cells is variable in different fish species according to their habitat and the intensity of light in it. The function of the rods is to detect the light, and that of the cones is to distinguish the wavelengths, i.e., color [59]. The current findings revealed a significant increase in cone cell densities in the ventral region compared to the dorsal retina, suggesting an adaptation for enhanced acute vision upwards. Comparable patterns have been observed in Salmon and *Poecilia reticulata* [60,61]. Kunz and Wise [61] noted that since fish primarily utilize cone vision for feeding, and considering Poecilia’s surface feeding behavior, the ventral retina is primarily dedicated to food detection and capture. The cones’ density can denote the level of sensation of motion in all directions [62]. It also helps migration over long distances [63]. The duplex retinas of many fish have variable areas of specialization [64]. For instance, the temporal area has closely packed cones, with rare rods, properly positioned along the primary feeding axis to receive enough light. A similar area is caudoventral in pelagic feeders, such as herring, which looks above and forward for food. It is caudal in the retina of horizontal feeders like sailfish (*Istiophorus*) and caudodorsal in bottom feeders such as sea bream (*Sparus*). A fossa with a high number of cones is present in seahorses (Hippocampus) and pipefish (*Syngnathus*). These specialized areas are not only found in fish. For example, there is a horizontal belt of cones in the amphibious mudskipper retina (*Periophthalmus*) to detect items close to the ground where both food and potential predators may be found [64].

Photoreceptors are specialized neurons with high metabolic activity. In the current study, the high staining response of the cones and rods for iron hematoxylin in Molly fish verified this, indicating the massive presence of mitochondria. Moreover, our histochemical analysis indicated that the IPL and OPL of the retina showed positive reactions for alcian blue. These results agree with the findings in frogs [65,66] and red-tail sharks [65,66], where this staining reaction indicates that the dye has bound to sulfate groups that are found in intracellular proteoglycans associated with membranes, such as those present in synaptic vesicles. These groups may serve as Ca^2^ binding sites or preserve other ionic equilibriums. These groups may also contribute to the preservation of the low pH that characterizes the lysosomal and endocytic compartments, as well as at least some classes of synaptic vesicles.

The melanin granules in the pigment epithelium, along with cones and rods, undergo circadian retinomotor motions [67]. Teleosts demonstrate the presence of the glycolytic enzyme lactate dehydrogenase in retinal photoreceptors and the optical regions of the brain, which could play a vital role in the regeneration of visual pigments [68]. Furthermore, teleost fish have the ability to regenerate neural tissue following damage. Cells that are damaged undergo apoptosis, clearing debris and paving the way for the creation of new neurons [9].

The present electron microscopy results indicated that the retina of Molly fish contained neuronal elements, such as horizontal, amacrine, bipolar, and glia cells, such as Müller cells. Amacrine cells serve as horizontal lines of communication for visual stimuli. Amacrine, bipolar, horizontal, and ganglion neurons are responsible for connecting and integrating photoreceptors with afferent neurons [67]. Ganglion cells play an essential role in sending collective messages to the optic nerve and brain. The signals from the photoreceptors are transmitted inward by the bipolar cells [68]. The current study showed that some processes from horizontal cells extend horizontally near the ONL and serve as lines of communication between the photoreceptors.

The inner nuclear layer contains the majority (75%) of the ganglion cells in lampreys [69], while the majority of ganglion cells in elasmobranchs and teleosts are located within the ganglionic layer, with only a small percentage “displaced” to the INL. There is still more research needed on the evolution of the inner retina in fishes [46].

The current immunohistochemical results indicated a strong GFAP expression within the OPL of the retina of Molly fish. These cells were identified as astrocytes because of their location and their unique cell processes [70]. The GFAP immunoreactive fibers within the optic nerve might be from optic nerve astrocytes similar to those described in carp (*Cyprinus carpio*), which reacted with the monoclonal antibodies of porcine GFAP [71].

GFAP immunoreactive Müller cells were identified by their distribution and morphology within the inner nuclear layer [70]. In the current study, Müller cells were also encountered in the outer zone of the inner plexiform layer. The GFAP immunoreactive puncta between inner segments of the cones and rods are mostly the microvilli from Müller cells [72,73]. Moreover, thin branches extended from the Müller cells in the ganglion layer. Most of these branches are directed towards the inner limiting membrane layer, penetrating the NFL to form the ILM by the endfeets of Müller cells [73]. We also found GFAP immunoreactive thick cell processes which appeared next to the ganglion cell layer before their endfeets.

Müller cell endfeets are essential for ionic balance in the retina [74]. A perfect exchange of nutrition throughout the retina is achieved by the expanded surface area resulting from the extensive system of Müller cell end-feet and the ILM. Müller glia have additional functions in zebrafish. They act as multipotent retinal stem cells, developing and regenerating retinal neurons [74,75]. Moreover, teleost Müller glia provide guidance to the continuously generated rod photoreceptors, from rod-specific progenitors. They use the radial fibers of Müller cells as scaffolds to reach their natural habitat in the photoreceptor layer [76].

While both Molly fish (*Poecilia sphenops*) and guppies (*Poecilia reticulata*) belong to the same family (Poeciliidae) and share similarities in their retinal structure with diurnal fish, there are hypothesized differences based on their specific adaptations and evolutionary history. The present study demonstrates that the density of rods and cones in the retina differs between Molly fish and the previously studied guppies [61], potentially reflecting disparities in their visual capabilities and ecological niches. Species-specific variations in rod and cone densities could influence differences in visual sensitivity or acuity. Furthermore, the current study reveals that retinal glial cells in Molly fish express GFAP, a marker that has not been examined in guppies in the literature. The expression patterns of glial cells, such as Müller cells and astrocytes, may differ between Molly fish and guppies. Although both species may have similar cell types in their retinas, the extent or distribution of glial fibrillary acidic protein (GFAP) expression could vary. To ascertain specific differences between the retinas of Molly fish and guppies, comparative studies focusing on the retinal molecular biology and visual acuity of both species would be necessary. Such studies could provide insights into the unique adaptations and visual strategies employed by these closely related fish species.

## 5. Conclusions

This study marks the first comprehensive record of the retinal structure of Molly fish, shedding light on their visual anatomy. This study delves into the retinal composition of Molly fish, revealing a square mosaic pattern of cones, including double cones and two variants of single cones. The presence of cone pedicles and rod spherules in the outer plexiform layer highlights intricates synaptic connectivity. Müller cells, expressing GFAP, extend across retinal layers, aiding structural support and metabolic regulation. Astrocyte cell processes expressing GFAP are observed in both plexiform layers, indicating their role in retinal homeostasis. The sophisticated retinal structure suggests robust photopic visual capabilities in Molly fish. The mosaic arrangement of photoreceptors and distinct GFAP expression patterns offer insights into adaptive strategies. These findings contribute to our understanding of retinal diversity among fish species and hint at future investigations into functional significance.

## Figures and Tables

**Table 1:** The mean density of cones and rods in the dorsal, temporal, ventral, and nasal regions of the retina in *Poecilia sphenops*.

	Dorsal	Ventral	Temporal	Nasal
Cones				
Mean	7143 ^c^	8786 ^a^	7370 ^bc^	6733 ^cd^
Std. Error	±144.9	±169.3	±92.68	±90.21
Rods				
Mean	6112 ^a^	5832 ^ab^	5679 ^abc^	4932 ^d^
Std. Error	±99	±96	±107	±126

^a,b,c,d^ Means in the same row with different superscripts are significantly different at *p* < 0.05 (one-way ANOVA followed by Tukey’s multiple range test).

## Data Availability

The data presented in this study are available on request from the corresponding author.
